# High rate of uncontrolled hypertension among adults receiving integrated HIV and hypertension care with aligned multi‐month dispensing in Malawi: results from a cross‐sectional survey and retrospective chart review

**DOI:** 10.1002/jia2.26354

**Published:** 2024-09-18

**Authors:** Hannah S. Whitehead, Khumbo Phiri, Pericles Kalande, Joep J. van Oosterhout, George Talama, Sam Phiri, Corrina Moucheraud, Agnes Moses, Risa M. Hoffman

**Affiliations:** ^1^ Department of Medicine David Geffen School of Medicine at UCLA Los Angeles California USA; ^2^ Partners in Hope Lilongwe Malawi; ^3^ School of Global and Public Health Kamuzu University of Health Sciences Lilongwe Malawi; ^4^ Department of Public Health Policy and Management School of Global Public Health at NYU New York City New York USA

**Keywords:** HIV, hypertension, integrated care, Malawi, sub‐Saharan Africa, multi‐month dispensing

## Abstract

**Introduction:**

People living with HIV have high rates of hypertension. Integrated HIV and hypertension care with aligned multi‐month dispensing of medications (MMD) could decrease the burden of care for individuals and health systems. We sought to describe hypertension control and evaluate its association with different durations of MMD among Malawian adults receiving integrated care with aligned dispensing of antiretroviral therapy (ART) and antihypertensive medication.

**Methods:**

We conducted a cross‐sectional survey and retrospective chart review of adults (≥18 years) receiving integrated HIV and hypertension care on medications for both conditions for at least 1 year, with aligned MMD at seven clinics in Malawi. Data were collected from July 2021 to April 2022 and included socio‐demographics, clinical characteristics, antihypertensive medications and up to the three most recent blood pressure measurements. Bivariate analyses were used to characterize associations with hypertension control. Uncontrolled hypertension was defined as ≥2 measurements ≥140 and/or ≥90 mmHg. Chart reviews were conducted for a random subset of participants with uncontrolled hypertension to describe antihypertensive medication adjustments in the prior year.

**Results:**

We surveyed 459 adults receiving integrated care with aligned dispensing (58% female; median age 54 years). Individuals most commonly received a 3‐month aligned dispensing of ART and antihypertensive medications (63%), followed by every 6 months (16%) and every 4 months (15%). Hypertension control was assessed in 359 respondents, of whom only 23% had controlled hypertension; 90% of individuals in this group reported high adherence to blood pressure medications (0−1 missed days/week). Control was more common among those with longer aligned medication dispensing intervals (20% among those with 1‐ to 3‐month dispensing vs. 28% with 4‐month dispensing vs. 40% with 6‐month dispensing, *p* = 0.011). Chart reviews were conducted for 147 individuals with uncontrolled hypertension. Most had high self‐reported adherence to blood pressure medications (89% missing 0−1 days/week); however, only 10% had their antihypertensive medication regimen changed in the prior year.

**Conclusions:**

Uncontrolled hypertension was common among Malawian adults receiving integrated care with aligned MMD and was associated with shorter refill intervals and few antihypertensive medication escalations. Integrated care with aligned MMD is promising, but further work is needed to understand how to optimize hypertension outcomes.

## 1 INTRODUCTION

Due to effective antiretroviral therapy (ART), people living with HIV (PLHIV) in low‐ and middle‐income countries are living longer and developing non‐communicable diseases (NCDs) associated with ageing, including hypertension, cardio‐ and cerebrovascular disease, chronic kidney disease and cancers [1, 2]. Additionally, the current global first‐line ART regimen contains dolutegravir, which is highly effective and well‐tolerated, but has been associated with weight gain and incident hypertension, especially in Black Africans [3, 4, 5]. Given the growing need to effectively manage NCD comorbidities in PLHIV, the World Health Organization now recommends integrating diabetes and hypertension care with HIV services, recognizing that integrated care could expand access to care and improve control of these common comorbidities [6]. Integrated care models throughout sub‐Saharan Africa (SSA) have been shown to be feasible, acceptable and cost‐effective [7, 8, 9].

There has been widespread scale‐up of multi‐month dispensing (MMD) of ART in Africa and this approach is endorsed in World Health Organization guidelines [6]. This model of care involves extending ART dispensing to provide a 3‐ or 6‐month supply of medications for stable clients [10]. MMD has been highly acceptable [11, 12], has non‐inferior HIV outcomes [13, 14] and reduces health system burden [15], but it has raised new challenges and questions about HIV integration with NCD care. Individuals living with HIV‐NCD comorbidities will not see the benefits of MMD for ART (e.g. reduced burden of care, reduced costs and time savings), if they are required to have frequent clinic visits for NCD care. Additionally, there are no data about whether MMD would improve NCD outcomes as it does for HIV outcomes. Aligned MMD of medications for both HIV and NCDs among people with multi‐morbidities could decrease the burden of care for these individuals, improve their clinical outcomes and have benefits for constrained health systems.

Malawi has a significant double burden of HIV and hypertension, with an adult HIV prevalence of 8.9% [16] and hypertension prevalence of approximately 30% (in the general population) [17]. Our previous prospective cohort study in Malawi found hypertension to affect nearly one in four PLHIV, with low rates of blood pressure control (19%) over 1 year of follow‐up [18]; however, these data were collected from a single facility prior to widespread integration of HIV‐NCD care and MMD for ART in Malawi, limiting our ability to explore alignment of MMD for HIV and hypertension in integrated clinic settings. Therefore, we sought to describe dispensing intervals among Malawian individuals receiving integrated HIV and hypertension care with aligned dispensing in the context of a broad scale‐up of 3‐ and 6‐month ART dispensing. We also explored the relationship between aligned MMD and hypertension control.

## 2 METHODS

Data were collected as part of a study of hypertension preferences using a discrete choice experiment with 1000 adults (18+ years), half living without HIV and half with HIV [19]. The parent study was conducted at 14 clinics in geographically representative districts of Malawi.

For this sub‐study, we included seven of the 14 facilities where integrated care was provided for HIV and hypertension, defined as clinical encounters in which the same provider performs the clinical assessment and dispenses medications for both conditions at the same visit (at the same time). Data were collected between July 2021 and April 2022. The seven facilities were in Central and Southern Malawi (four urban and three rural), all providing free HIV care, including medications (funded by the government with assistance from PEPFAR/USAID). Six of the seven facilities also provided hypertension care and medications free of charge (funded through the Malawi government). One site was affiliated with the Christian Health Association of Malawi network and charged for hypertension care and medications. At the time of this study, HIV guidelines included 3‐month ART dispensing for stable clients (those with viral suppression in the prior year and no active clinical issues requiring frequent follow‐up); however, under the COVID‐19 pandemic (early 2020 onwards), 6‐month ART dispensing was widely scaled in Malawi. New HIV clinical management guidelines released in January 2022 formally recommended 6‐month dispensing for stable ART clients [20, 21].

At the time of data collection, there was no formal guidance for antihypertensive medication refill/dispensing frequency and stocks of antihypertensive medications were known to vary by facility. Therefore, facility surveys were performed during the period of data collection to understand how drug supply might influence the study's results. These surveys showed that all participating facilities had a continuous stock of first‐line therapy (diuretic), 63% had consistent supplies of second‐line medications (amlodipine and/or nifedipine) and all had adequate supplies of third‐line medications (captopril and/or enalapril).

Eligible participants for this study were adults (≥18 years) on ART and antihypertensive medications during the previous year with aligned dispensing (including 1‐month dispensing). Pregnant and breastfeeding women were excluded from the parent study and, therefore, not included in this analysis. Alignment was determined by asking participants about the frequency of integrated ART‐hypertension visits and the frequency of any additional visits specifically for antihypertensive medication refills over the prior 12 months. Those who had extra visits for antihypertensive medication refills were considered not aligned and excluded from the study.

The target sample was 500 participants, and was determined by the parent study, which sought to enrol 500 individuals living with HIV and hypertension and was powered for the primary discrete choice experiment (DCE) outcome. Because of the exploratory nature of the study, no power calculations were performed. For the parent study, convenience sampling was used to select participants as they waited for care in ART and NCD clinics.

During the time period of the study, treatment of hypertension in Malawi was based on national treatment guidelines [22] that defined stepwise escalation of therapy for blood pressure values remaining elevated (≥140 or ≥90 mmHg) despite current therapy. First‐line treatment was a thiazide diuretic, with the addition of the following in a stepwise manner: calcium channel blocker, followed by an angiotensin‐converting enzyme inhibitor (ACEI), followed by a beta blocker.

### 2.1 Uncontrolled hypertension definition

Individuals were included in the analysis of hypertension control if they had either two or three visits with a blood pressure recorded in the prior 12 months. If more than three blood pressures were recorded, only the most recent three blood pressures were collected and used for this analysis. Uncontrolled hypertension was defined as either both readings (if two available), or two of the three readings with a systolic blood pressure ≥140 mmHg and/or a diastolic blood pressure ≥90 mmHg. Recognizing that changes made in medications during the year may be best reflected by the final blood pressure recording, we performed a sensitivity analysis using only this value to define control. For this analysis, if the final blood pressure (most proximate to the date of data collection) was <140/90, the respondent was considered controlled. If the final blood pressure was ≥140/90, they were considered uncontrolled. All analyses were repeated using this alternative definition of control.

### 2.2 Surveys

Surveys were administered in the local language (Chichewa), in a private space at the clinic, and collected data on socio‐demographic characteristics, duration living with hypertension and duration on antihypertensive medications, adherence to antihypertensives (asked as number of days per week blood pressure medications are missed in an average month), self‐report of a diagnosis of diabetes or heart disease and frequency of visits for care (to determine the interval of aligned MMD). Due to low literacy in the population, each survey question was read out loud by the research assistant, and participants’ responses were recorded in a tablet.

### 2.3 Medical record review

Medical records were reviewed to record the three most recent blood pressures in the prior 12 months and for all antihypertensive medications prescribed in the prior 12 months. If more than one blood pressure was recorded at the same visit, we used an average of the blood pressures recorded. A random subset of participants with uncontrolled hypertension was selected for a more detailed chart review to collect antihypertensive medication name and quantities dispensed at each visit (separately for each medication) over the prior year. A sample size of 150 was selected based on feasibility within the funding and timeframe available and to allow for an exploration of medication adjustments among respondents with uncontrolled hypertension. Dose adjustments were not captured because the Malawi Standard Treatment guidelines do not recommend titrating thiazide (first‐line therapy) and focus on adding new classes for persistent hypertension, with the exception of ACEIs, which may be titrated [22].

Data were collected using the SurveyCTO mobile application on Android tablets. Informed consent was obtained prior to commencing data collection. This study was embedded into a larger study of hypertension care preferences among Malawian adults with and without HIV and was a specific module incorporated for PLHIV. The study was approved by the Malawi National Health Science Research Committee (NHSRC #20/07/2577) and the Institutional Review Board at the University of California, Los Angeles (IRB # #20‐001856).

### 2.4 Data analysis

Descriptive and bivariate analyses (using chi‐square, Fisher's exact and *t*‐tests) were used to describe the sample and explore associations of socio‐demographics, clinical characteristics and aligned MMD with hypertension control. Multivariable logistic regression was performed on the outcome of controlled blood pressure evaluating MMD alignment interval, clinical and socio‐demographic variables. Data cleaning and statistical analyses were conducted in Stata 17.0. Characterization of hypertension medication changes over 1 year was conducted in Excel.

## 3 RESULTS

Of the 500 PLHIV in the parent study, 459 were receiving integrated care with aligned MMD and were included in our sample; 58% were female and the median age was 54 years (IQR 48−60). Participants had been on ART for a median of 10.5 years, with nearly all (97%) being on tenofovir disoproxil fumarate/lamivudine/dolutegravir; 98.8% of those with a viral load recorded in the past 2 years (431/459) were suppressed (<200 copies/ml). Participants had been on antihypertensive medications for a median of 5.0 years, and approximately 8% and 2% self‐reported having diabetes or heart disease, respectively. Women were more likely to be younger and unmarried, but were otherwise similar to men. The most common aligned MMD interval was every 3 months (63%), followed by every 6 months (16%) and every 4 months (15%). Approximately 6% of participants were receiving aligned dispensing with visits every 1 or 2 months (Table 1).

**Table 1:** Sample characteristics of Malawian adults receiving integrated HIV and hypertension care with aligned dispensing of ART and antihypertensives (*n* = 459)

	Male (*n* = 194)		Female (*n* = 265)		Total (*n* = 459)	
**Age**	***N***	**%**	***N***	**%**	***N***	**%**
Median (IQR)	57	(49−62)	53	(48−59)	54	(48−60)
**Marital status**						
Married or long‐term partner	174	89.7%	130	49.1%	304	66.2%
Not married	20	10.3%	135	50.9%	155	33.8%
**Employment status**						
Working (formally or informally)	178	91.8%	232	87.6%	410	89.3%
Not working	16	8.3%	33	12.5%	49	10.7%
**Self‐rated socioeconomic status**						
Step 1 (Poorest)	42	21.7%	50	18.9%	92	20.0%
Step 2	72	37.1%	102	38.5%	174	37.9%
Step 3	72	37.1%	94	35.5%	166	36.2%
Step 4	8	4.1%	19	7.2%	27	5.9%
Step 5	0	0.00%	0	0.00%	0	0.00%
Step 6 (Wealthiest)	0	0.00%	0	0.00%	0	0.00%
**Health characteristics**						
Median years on ART (IQR)	11	(6−15)	11	(7−15)	11	(6−15)
Median years with hypertension (IQR)	5	(3−8)	5	(3−9)	5	(3−9)
Median years on hypertension meds (IQR)	4	(3−7)	5	(3−8)	5	(3−7)
Have diabetes^a^	18	9.3%	19	7.2%	37	8.1%
Have heart disease^a^	1	0.5%	8	3.0%	9	2.0%
**ART regimen**						
TLD	189	97.4%	256	96.6%	445	97.0%
Other^b^	5	2.6%	9	3.4%	14	3.1%
Viral load <200 copies/ml^c^	182	98.9%	244	98.8%	426	98.8%
**Aligned dispensing interval**						
Monthly or shorter	6	3.1%	12	4.5%	18	3.9%
Every 2 months	1	0.5%	8	3.0%	9	2.0%
Every 3 months	132	68.0%	158	59.6%	290	63.2%
Every 4 months	28	14.4%	39	14.7%	67	14.6%
Every 6 months	27	13.9%	48	18.1%	75	16.3%

Abbreviations: ART, antiretroviral therapy; IQR, interquartile range; TLD, tenofovir disoproxil fumarate/lamivudine/dolutegravir.

^a^ Based on self‐report.

^b^ Nine on dolutegravir with alternative nucleoside reverse transcriptase (NRTI) backbone; two on two‐drug regimens; two on protease‐inhibitor‐based regimens with boosted atazanavir and one on efavirenz with an NRTI backbone.

^c^ Viral load available in the prior 2 years 431/459.

### 3.1 Hypertension control

**Figure 1:**
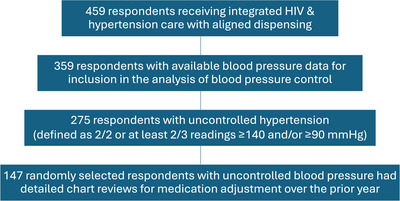
Flow chart of participants included in subsets for analysis

One hundred individuals were excluded from the analysis of hypertension control because they did not have three blood pressures documented in their medical records over the prior year. Figure 1 demonstrates how the sample contributed to the aims of hypertension control (*n* = 359) and detailed chart review to describe medication adjustments among those with uncontrolled hypertension (*n* = 147). Twenty‐three percent of individuals (*n* = 84) had controlled hypertension. Twenty‐one individuals (5.8%) reported not taking any medications in the prior 30 days, and of the remaining participants (*n* = 338), the majority (95%) reported high adherence to antihypertensives (0−1 days per week with no doses taken, on average). Being female and currently employed were associated with controlled hypertension (27.5% of females vs. 17.8% of males, *p* = 0.031; 25.3% of currently working individuals vs. 9.3% of those not working, *p* = 0.02) (Table 2). Individuals who were on two or more antihypertensive medications were more likely to have controlled hypertension than those on only one medication, though this difference was not statistically significant (27.0% controlled among those on two or more medications vs. 19.4% among those on one medication, *p* = 0.091). Blood pressure control improved as the duration of aligned MMD increased from 3 to 4 to 6 months (18.3% with 3MMD, 27.9% with 4MMD, 39.5% with 6MMD). Control was 35.0% in the 1−2 MMD group, although interpretation is limited by the small sample size in this group (*n* = 20). In a multivariable regression model that included MMD alignment interval, age, gender, employment, duration with hypertension and number of hypertension medications, only female gender (adjusted OR 1.8, 95% CI 1.1−3.1, *p* = 0.03) and current employment (adjusted OR 3.7, 95% CI 1.2−11.1, *p* = 0.02) were significantly associated with blood pressure control.

**Table 2:** Factors associated with blood pressure control (*n* = 359)

		Uncontrolled hypertension^a^ (*n* = 275)		Controlled hypertension^a^ (*n* = 84)		
	Total *N*	*N*	%	*N*	%	*p*‐value
**Dispensing frequency**						
Monthly or 2MMD	20	13	65.0%	7	35.0%	0.008
3MMD	235	192	81.7%	43	18.3%	
4MMD	61	44	72.1%	17	27.9%	
6MMD	43	26	60.5%	17	39.5%	
**Gender**						
Male	152	125	82.2%	27	17.8%	0.031
Female	207	150	72.5%	57	27.5%	
**Age**						
Median (IQR)		53	(48−59)	55	(50−61)	0.237
**Self‐reported socioeconomic status**						
Step 1 (Poorest)	65	50	76.9%	15	23.1%	0.84
Step 2	141	111	78.7%	30	21.3%	
Step 3	131	97	74.1%	34	26.0%	
Step 4	22	17	77.3%	5	22.7%	
Step 5	0	0	0%	0	0%	
Step 6 (Wealthiest)	0	0	0%	0	0%	
**Employment status**						
Working	316	236	74.7%	80	25.3%	0.02
Not working	43	39	90.7%	4	9.3%	
**Number of HTN meds**						
1 medication	170	137	80.6%	33	19.4%	0.091
2 or more medications	189	138	73.0%	51	27.0%	
**Self‐reported adherence to HTN medications (days missed/week in an average month)** ^b^						
0‐10−1 days	322	242	75.2%	80	24.8%	0.424
2−3 days	12	10	83.3%	2	16.7%	
4 or more days	4	4	100%	0	0%	
**Years with HTN**						
1−3 years	124	95	76.6%	29	23.4%	0.709
4−9 years	152	119	78.3%	33	21.7%	
10+ years	83	61	73.5%	22	26.5%	
**Years on ART**						
Less than 3 years	30	23	76.7%	7	23.3%	0.586
4−9 years	110	88	88.0%	22	20.0%	
10+ years	219	164	74.9%	55	25.1%	

Abbreviations: ART, antiretroviral therapy; HTN, hypertension; IQR, interquartile range; MMD, multi‐month dispensing.

^a^ Uncontrolled blood pressure was defined as 2/2 or at least 2/3 blood pressure readings in the prior year (based on the most recent three readings in the chart) with a systolic blood pressure ≥140 mmHg and/or a diastolic blood pressure ≥90 mmHg.

^b^ Excludes 21 people not taking any medications in the prior 30 days.

In a sensitivity analysis using only the most recent blood pressure in the chart to define controlled blood pressure (and using the same threshold of <140/90 mmHg), we found similar results: 95 participants (26.4%) were controlled compared to 84 (23.4%) using the three most recent blood pressures. Additionally, the analyses of factors associated with blood pressure control were similar using this definition.

### 3.2 Antihypertensive medication regimens

The most common regimen was hydrochlorothiazide monotherapy (*n* = 152, 42.3%), followed by a combination of hydrochlorothiazide and amlodipine (*n* = 66, 18.1%). Uncontrolled hypertension was most common among those on hydrochlorothiazide monotherapy (84.2%) and least common in those on a combination of hydrochlorothiazide and amlodipine (63.6%) (Table 3).

**Table 3:** Common antihypertensive medication regimens overall and by hypertension control status (*n* = 359)

	Uncontrolled hypertension (*n* = 275)		Controlled hypertension (*n* = 84)		Total (*n* = 359)	
Current regimen	*N*	%	*N*	%	*N*	%
**HCTZ monotherapy**	128	84.2%	24	15.8%	152	42.3%
**HCTZ + amlodipine**	42	63.6%	24	36.4%	66	18.4%
**HCTZ + amlodipine + enalapril**	33	80.5%	8	19.5%	41	11.4%
**HCTZ + enalapril**	26	72.2%	10	27.8%	36	10.0%
**All other regimens**	46	71.9%	18	28.1%	64	17.8%

Abbreviation: HCTZ, hydrochlorothiazide.

### 3.3 Antihypertensive medication adjustments in participants with uncontrolled hypertension

A sample of 147 participants with uncontrolled hypertension had medical records reviewed for all antihypertensive medications within the past 12 months. The participants included in this analytic subset were similar to the overall cohort (50.3% female, median age 52 years, median duration on antihypertensive medications 5 years). Fourteen participants (9.5%) reported not taking medications in the month prior, and among the remainder (*n* = 133), 98.5% reported missing medications 0−1 day per week, on average. The majority of these (*n* = 132, 89.8%) were prescribed the same antihypertensive medications at all clinic visits in the past year despite having two or more blood pressures ≥140 or ≥90 mmHg. Fifteen individuals (10.2%) had changes to their antihypertensive medications over the year. Most of these (*n* = 14) were receiving 3‐monthly or longer aligned dispensing intervals, including six individuals who were receiving 6‐monthly aligned dispensing. Only one individual with an adjustment was on a monthly dispensing schedule. Medication adjustments included escalation in therapy (*n* = 10), medication class change (*n* = 2) and medication discontinued with no other change (*n* = 3). Of the 10 individuals with escalation, all were consistent with recommended treatment guidelines.

## 4 DISCUSSION

Uncontrolled hypertension was common in this sample of Malawian adults receiving integrated HIV and hypertension care with aligned MMD of medications for both conditions. Our prevalence of hypertension control (23%) is consistent with that of our previous cohort of Malawian adults on ART [18]. We found control rates to be lower than studies from Africa of adults without HIV, which range from approximately 40% to 50% [23, 24].

Hypertension control was less common among individuals receiving shorter aligned MMD for ART and antihypertensives (≤3 MMD). Importantly, these visits were not explained by medication adjustments, as the majority of individuals had no medication escalations during the year, despite having at least two blood pressures in the elevated range during this same time frame. Hypertension control was modestly better in those with longer dispensing intervals; particularly for those receiving 6 months of medications. It is possible that these “most stable” clients were preferentially given 6‐month dispensing because of their clinical stability. It is also possible that longer MMD reduced the burden of care and allowed clients to have better adherence to antihypertensive medications, consistent with documented benefits of MMD for ART [25, 26, 27, 28, 29, 30]. Our findings are similar to an observational study from Uganda that assessed integrated 3‐month dispensing of ART and antihypertensive medications, and found that hypertension control significantly increased over 12 months of follow‐up [31]. Of note, 3‐ and 6‐month MMD of antihypertensive medications for adults with controlled blood pressure is recommended by WHO guidelines for the treatment of hypertension [32].

Malawi has national guidelines on the management of hypertension [31], which outline stepwise additions of antihypertensive classes for persistently elevated blood pressure. Our data suggest that these guidelines were not universally followed, as the majority of clients remained on the same regimen throughout the year despite elevated blood pressures and high self‐reported adherence to antihypertensive medications. We do not have data from clinicians to understand the reasons for their lack of antihypertensive escalation. Clinical or therapeutic inertia (lack of timely adjustment of medications to achieve improved hypertension control) may be a contributing factor and has been reported from diverse settings, including Africa [33, 34, 35]. Self‐reported antihypertensive medication adherence is subject to reporting bias, and providers may have been aware of adherence challenges that participants did not disclose to the study team. The supply chain for antihypertensive medications in Malawi is challenging [36, 37], and this may play a role in limiting escalations and switches. However, facility surveys done contemporaneously to this project showed that all participating facilities had a continuous stock of first‐line thiazide, almost two‐thirds (63%) had consistent supplies of second‐line medications (amlodipine and/or nifedipine) and all had adequate supplies of third‐line medications (captopril and/or enalapril), making supply constraints less likely to be the sole reason for our findings.

An alternative explanation for the lack of escalation of antihypertensive treatment when indicated may be that clinicians providing integrated ART and NCD care are required to manage individuals with multiple comorbidities. HIV care has significant clinical care and reporting requirements, and HIV may be prioritized in integrated care settings, limiting the clinicians’ ability to focus on hypertension. Past research also indicates a lack of facility and clinician capacity and readiness for management of NCDs in Malawi and other countries in SSA [38, 39, 40]; and while Malawi's guidelines recommend a stepped escalation protocol, they do not clearly indicate when a client's antihypertensive treatment should be escalated. Clinician training and coaching, along with disseminating more specific instructions and treatment algorithms, could help improve hypertension management and clinical outcomes.

Over three‐quarters of this sample of adults had uncontrolled hypertension, while over 98% had viral loads less than 200 copies/ml. Our data indicate that within integrated HIV‐NCD services, high rates of treatment success with HIV do not necessarily translate to similarly good outcomes for hypertension, even in the context of alignment of medications for both conditions that are available free of charge. There has been a significant push for integration of HIV with other NCD services in hopes that integration alone would result in NCD disease control; however, our data do not support this hypothesis. Another study from Malawi showed that despite integrated care for HIV and hypertension at a large, urban clinic, only approximately 30% of clients had controlled hypertension after 6 months of follow‐up [36]. There are numerous potential reasons why integration is “not enough,” including low client knowledge about hypertension (relative to HIV); the fact that, in certain clinics, hypertension medications are available only for a cost; because of adherence challenges with antihypertensive medications due to pill burden and competing life priorities; and related to medication side effects, which have been reported in other studies of hypertension treatment in Africa [41]. It is possible that monotherapy is not adequate to achieve blood pressure control for PLHIV and further studies should explore two agents as initial therapy for hypertension in this context. The WHO hypertension treatment guidelines recommend initiation of two drugs (preferentially as a single pill combination) for adults requiring pharmacologic treatment of hypertension [32]; however, while these single pill combinations are increasingly available globally [42], they are not currently available in Malawi. Poor antihypertensive medication quality may also be a factor in low rates of uncontrolled hypertension in the region [43]. Ultimately, while integrated care with aligned MMD holds promise for addressing the increasing burden of comorbidities among ageing PLHIV, there is mounting data that additional interventions are needed to optimize the control of hypertension.

### 4.1 Limitations

We acknowledge several limitations of this analysis. The cross‐sectional design of our study precludes conclusions about causality, and we thus cannot assess whether individuals with poor blood pressure control were intentionally seen more frequently for more intensive management or that those selected for MMD had better blood pressure trends over time or were felt to be more likely to have treatment success—however, the lack of medication adjustments in these clients despite multiple high blood pressures suggests other factors account for decisions about MMD and follow‐up interval. We did not power our study for a multivariable analyses and larger studies are needed to confirm our results. We did not collect doses of medications and, therefore, cannot account for dose escalations. This study was performed during a phase of the COVID‐19 pandemic when surges were occurring and this may have influenced provider behaviour around alignment and MMD—in particular, providers may have given larger quantities of medications despite suboptimal blood pressure control to limit the need for return visits. COVID‐19‐related challenges may have also been the reason that 100 of the respondents did not have sufficient blood pressure data to be included in the analysis of blood pressure control. The exclusion of these individuals is an important limitation.

## 5 CONCLUSIONS

Uncontrolled hypertension was very common among Malawian adults receiving integrated HIV and hypertension care with aligned MMD. Uncontrolled hypertension was associated with high self‐reported antihypertensive medication adherence and shorter refill intervals in the unadjusted analysis, but without antihypertensive escalations over the timeframe reviewed. In Malawi and similar settings, integrated care with aligned MMD is promising for hypertension and other NCD care, but more work is needed to understand factors associated with hypertension control and to introduce feasible interventions for effective hypertension management. Future research should aim to understand how to improve rates of blood pressure control among a larger population from multiple perspectives, including from clients, clinicians and Ministry of Health leaders.

## COMPETING INTERESTS

None of the authors have competing interests.

## AUTHORS’ CONTRIBUTIONS

RMH, JJvO, CM and SP designed the study; HSW performed data analyses with support from RMH and CM and took the lead in manuscript writing and revisions; KP and PK contributed to data collection processes and study implementation, and provided interpretation of data; AM provided oversight of the study; all authors including GT and JC reviewed and edited the manuscript.

## FUNDING

This work was made possible by support from the NIH Fogarty International Center grant 5R21TW011691.

## Data Availability

After completion of the parent study and all sub‐studies, anonymized data can be shared with permission from the study PIs.
